# The effect of neoadjuvant therapy on PD-L1 expression and CD8+lymphocyte density in non-small cell lung cancer

**DOI:** 10.1038/s41379-022-01139-y

**Published:** 2022-08-01

**Authors:** Philipp Zens, Corina Bello, Amina Scherz, Michael von Gunten, Adrian Ochsenbein, Ralph A. Schmid, Sabina Berezowska

**Affiliations:** 1 Institute of Pathology, University of Bern, Bern, Switzerland; 2 Graduate School for Health Science, University of Bern, Bern, Switzerland; 3 Department of Medical Oncology, Inselspital, Bern University Hospital, Bern, Switzerland; 4 Pathology Länggasse, Bern, Switzerland; 5 Department of General Thoracic Surgery, Inselspital, Bern University Hospital, Bern, Switzerland; 6 Institute of Pathology, Lausanne University Hospital and University of Lausanne, Lausanne, Switzerland; 7 Present Address: Department of Anesthesiology, Hospital Grabs, Spitalstrasse 44, CH-9472 Grabs, Switzerland

**Keywords:** Prognostic markers, Predictive markers

## Abstract

PD-L1 expression is the routine clinical biomarker for the selection of patients to receive immunotherapy in non-small cell lung cancer (NSCLC). However, the application and best timing of immunotherapy in the resectable setting is still under investigation. We aimed to study the effect of chemotherapy on PD-L1 expression and tumor infiltrating lymphocytes (TILs), which is to date still poorly understood. Our retrospective, single-centre neoadjuvant cohort comprised 96 consecutive patients with NSCLC resected 2000–2016 after neoadjuvant therapy, including paired diagnostic chemo-naïve specimens in 53 cases. A biologically matched surgical cohort of 114 primary resected cases was included. PD-L1 expression, CD8 + TILs density and tertiary lymphoid structures were assessed on whole slides and correlated with clinico-pathological characteristics and survival. Seven/53 and 12/53 cases had lower respectively higher PD-L1 expressions after neoadjuvant therapy. Most cases (*n* = 34) showed no changes in PD-L1 expression, the majority of these harboring PD-L1 < 1% in both samples (21/34 [61.8%]). Although CD8 + TILs density was significantly higher after chemotherapy (*p* = 0.031) in resections compared to diagnostic biopsies, this might be due to sampling and statistical bias. No difference in PD-L1 expression or CD8 + TILs density was detected when comparing the neoadjuvant and surgical cohort. In univariable analyses, higher CD8 + TILs density, higher numbers of tertiary lymphoid structures but not PD-L1 expression were significantly associated with longer survival. Increased PD-L1 expression after neoadjuvant chemotherapy was not significantly associated with shorter 5-year survival, but the number of cases was very low. In multivariable analysis, only pT category and age remained independent prognostic factors. In summary, PD-L1 expression was mostly unchanged after neoadjuvant chemotherapy compared to diagnostic biopsies. The sample size of cases with changed PD-L1 expression was too small to draw conclusions on any prognostic value.

## Introduction

Lung cancer is the deadliest cancer worldwide, mainly explainable by the late diagnosis due to presentation in advanced stages (UICC/AJCC TNM stage III/IV)^1^.

For early-stage non-small cell lung cancer (NSCLC), surgery still offers the best chance of cure^2^. However, especially in nodal-positive patients, there is a high risk of recurrence and death. Since randomized trials have shown that additional neoadjuvant or adjuvant chemotherapy leads to better outcomes compared to resection only, it is generally accepted that patients with lymph node metastatic NSCLC should not receive surgery as a stand-alone treatment^3,4^. Adjuvant platinum-based chemotherapy offers a modest 5-year survival benefit of about 5% and is recommended for patients with completely resected early-stage, high-risk NSCLC – weighing the benefits and risks^5^. Perioperative therapeutic approaches are a hotly debated topic, with immunotherapy-based combinations and targeted treatments – in EGFR mutated NSCLC – dominating the current trial landscape. According to surgical outcomes from the phase III CheckMate 816 trial, the neoadjuvant combination of nivolumab and chemotherapy showed considerably lower rates of residual tumor compared with chemotherapy alone^6^. Primary results of the phase III global IMpower010 trial demonstrated a significant improvement in disease-free survival (DFS) comparing atezolizumab with best supportive care after adjuvant chemotherapy for resected stage IB – IIIA NSCLC. The greatest benefit was observed in patients with a tumoral programmed cell death 1 ligand 1 (PD-L1) expression ≥1%^7^.

Programmed cell death protein 1 (PD1) and its ligand PD-L1 belong to the costimulatory pathway of the adapted immune system^8^. Extensive studies have explained the hijacking of this regulatory pathway by different tumor entities including lung cancer^8^. PD1 is expressed on the surface of T cells, mediating inhibitory and stimulatory signals^8^. The overexpression of PD-L1 on the surface of tumor cells leads to evasion of an appropriate tumor-induced response of the immune system by T cell apoptosis and exhaustion^8^. The combination of immune checkpoint blockade and chemotherapy seems to be beneficial especially in patients with low levels of PD-L1 expressing tumor cells and ongoing trials are reporting positive results of this regimen in patients with resectable lung cancer^6,9^. However, the selection of appropriate patients is currently based only on PD-L1 expression in tumor tissue prior to medication, which is a suboptimal biomarker. More selective tools or, conceivably, a combination of multiple tumor immunity markers such as tumor mutational burden or CD8 + tumor infiltrating lymphocytes (TILs) are needed to predict response to treatment. This is highlighted by recent examples of immune checkpoint blockade (ICB) benefit irrespective of tumoral PD-L1 expression^10^. Furthermore, it is still unclear how classic neoadjuvant chemotherapy influences the tumor microenvironment and if it could promote therapeutic ICB. Regarding the neoadjuvant setting, several studies have reported dynamic changes, though without a clear trend of altered PD-L1 expression after chemotherapy (Table 1)^11–23^. These results contrast preclinical data substantiating an immunogenic effect to some chemotherapeutic agents and functional studies reporting the mechanisms involved in chemotherapy resistance and PD-L1 upregulation^14,19,24^.

**Table 1:** Currently published studies investigating the effect of neoadjuvant regimens on PD-L1 expression and the immune microenvironment.

study	year	number of patients (paired/PD-L1/CD8)	histology	neoadjuvant treatment	PD-L1 clone	PD-L1 evaluation	PD-L1 results			CD8 clone	CD8 evaluation	CD8 conclusion (change after neoadj. treatement)
							Conclusion (change after neoadj. treatment)	categories	Unchanged, *n* (%)			
Remark^11^	2016	21/122/122	LUSC/ LUAD/LCC	CT + CRT	E1L3N	TPS	no change	no	NA	SP16	density	no change
Sheng^12^	2016	32/32/0	LUSC/LUAD	CT	E1L3N	H-Score	decrease	additional <1% 1-<5% 5-<10% ≥10%	NA	NA	NA	NA
Song^13^	2016	76/76/0	LUSC	CT	NA	H-Score	increase	no	65 (85.5)	NA	NA	NA
Zhang^14a^	2016	30/92/92	LUSC/LUAD	CT	Abcam^b^	H-Score	NA	NA	NA	Abcam^b^	H-Score	NA
Fujimoto^15^	2017	35/35/34	LUSC/LUAD	CRT	28–8	TPS	decrease	<1% 1–49% ≥50%	15 (42.9)	C8/144B	SPS^c^	increase
Parra^16^	2018	0/112/112	LUSC/LUAD	CT	E1L3N^d^	TPS (automated, 5 hotspots)	increase	no	NA	C8/144B^d^	density (automated, 5 hotspots)	no change
Rojko^17^	2018	41/41/0	LUSC/LUAD/ NSCLC/SCLC	CT	SP142	TPS (3 TMA spots)	no change	1% 5% 10% 50%^e^	29 (70.7)	NA	NA	NA
Choe^18^	2019	33/43/43	LUSC/non-LUSC	CRT	22C3 SP263	TPS	no change	no	16 (48.5)	C8/144B	SPS^c^	increase
Fournel^19^	2019	39/39/39	LUSC/LUAD/ NSCLC/LCNEC	CT	E1L3N	TPS	increase	no	8 (20.5)	SP16	density (automated, selected spots)	no change
Guo^20^	2019	63/63/0	LUSC/LUAD/NSCLC/NEC	CT	22C3	TPS	increase	<1% 1–49% ≥50%	40 (63.5)	NA	NA	NA
Shin^21^	2019	86/86/0	LUSC/LUAD/NSCLC/LCC	CRT	E1L3N	TPS	increase	<1% 1–5% 5–50% ≥50%	40 (46.5)	NA	NA	NA
Yoneda^22^	2019	41/41/41	LUSC/LUAD	CT group CRT group	E1L3N	TPS	increase (CCRT) no change (CT)	no	NA	C8/144B	SPS	increase
Gaudreau^23^f	2021	0/82/82	LUSC/non-LUSC	CT	E1L3N^d^	NA	NA	NA	NA	C8/144B^d^	proportion (automated, 5 spots)	increase

*LUSC* lung squamous cell carcinoma, *LUAD* lung adenocarcinoma, *LCC* large cell carcinoma, *NSCLC* non-small cell lung carcinoma, *SCLC* small cell lung cancer*, LCNEC* large cell neuroendocrine carcinoma, *NEC* neuroendocrine carcinoma, *TPS* tumor proportional score, *SPS* stroma proportional score, *CRT* chemoradiotherapy, *CT* chemotherapy, ^a^comparison was made but not statistically assessed, ^b^rabbit anti-PD-L1/CD8 from Abcam, ^c^classified as ≤25%, 26–50%, >50% of nucleated cells in the stroma, ^d^multiplex immunofluorescence, ^e^additional classification in <1%, 1–5%, 6–10%, 11–20%, 21–30%, 31–40%, 41–50%, 51–60%, 61–70%, 71–80%, 81–90%, 91–100% TPS, ^f^follow-up study of Parra et al. with potential overlap of the cohorts, PD-L1 was stained but the results were not reported.

Here, we aimed to assess PD-L1 expression and CD8 + TILs density and their prognostic importance in a real-life cohort of patients with NSCLC resected after neoadjuvant chemo(radio)therapy compared to paired diagnostic samples and a biologically matched surgical cohort with primary resected advanced NSCLC.

## Material and methods

### Study population

This retrospective single center study was conducted on consecutive patients with NSCLCs, resected between January 2000 and December 2016 in the Department of thoracic surgery of the Inselspital and diagnosed at the Institute of Pathology, University of Bern. It includes a neoadjuvant cohort of cases resected after neoadjuvant (radio-)chemotherapy, as previously described^25^, and a biologically matched surgical cohort of primary resected cases of lung squamous cell carcinoma (LUSC) and lung adenocarcinoma (LUAD) at a locally advanced stage, defined by the presence of mediastinal lymph-node metastases (pN2)^26^. The cases were included according to pathology reports, validated and expanded by considering the clinical files of the Inselspital Bern (clinical data), cantonal cancer registry of Bern (survival data) and by contacting the general practitioners (clinical and survival data).

The initial neoadjuvant cohort consisted of 130 patients and was reduced to 118 patients after excluding tumors with neuroendocrine histology and patients not treated with neoadjuvant intention (flowchart in supplementary fig. S1). For PD-L1 and tumor microenvironment evaluation (CD8 + TILs and tertiary lymphoid structures, TLS), the cohort was further reduced to 96 respectively 95 patients due to insufficient residual tumor in the excluded case (Supplementary fig. S1). For one patient with a LUSC a second sample was evaluated for PD-L1 due to the incidental diagnosis of an independent small LUAD. Regarding pre-neoadjuvant therapy specimens, 86/118 patients had available diagnostic biopsies or cytology specimens, in 53 cases with sufficient tumor content for PD-L1 assessment and in 36 cases with adequate material for CD8 + TILs evaluation, excluding cytologies and lymph node biopsies without desmoplastic reaction. TLS were not evaluated in the pretherapeutic specimens.

The surgical cohort was biologically matched, meaning that the final tumor stage would have qualified for neoadjuvant therapy (pN2), had it been suspected preoperatively. It consisted initially of 115 cases including 60 patients with LUAD and 55 patients with LUSC. Finally, 114 patients were included for PD-L1 evaluation and 111 patients for CD8 + TILs and TLS evaluation (Supplementary fig. S1).

For harmonization, all cases were pathologically re-evaluated by SB and PZ and re-staged according to the current 8^th^ edition of the UICC TNM classification^26^. We only included the pathological TNM classification as assessed using the resection specimens. Additionally, the predominant growth pattern was assessed for primary resected LUAD cases according to the current 2021 World Health Organization criteria^27^. Table 2 summarizes the baseline characteristics of the cohorts including p-values of the statistical comparison if applicable. We did not statistically compare tumor stages (due to selection bias in the surgical cohort as described above), growth pattern (not assessable after neoadjuvant therapy) and neoadjuvant (radio)-chemotherapy (only applied to patients of the neoadjuvant cohort).

**Table 2:** Baseline characteristics of the neoadjuvant and surgical cohort.

	Neoadjuvant cohort	Surgical cohort	*p* value
	(*n* = 96)	(*n* = 114)	
*Age (median [IQR])*	63.50 [55.75, 70.00]	63.50 [58.00, 70.00]	0.418°
*Sex (%)*	***n* = 96 (%)**	***n* = 114 (%)**	0.553*
Female	28 (29.2)	38 (33.3)	
Male	68 (70.8)	76 (66.7)	
*Smoking status (%)*	***n*** = **83 (%)**	***n*** = **90 (%)**	0.429*
Never/ex-smoker	56 (67.5)	55 (61.1)	
Active smoker	27 (32.5)	35 (38.9)	
*Histology (%)*	***n*** = **96 (%)**	***n*** = **114 (%)**	0.678*
LUSC	46 (47.9)	54 (47.4)	
LUAD	47 (49.0)	60 (52.6)	
Other	3 (3.1)		
*Tumor size (median [IQR])*	3.20 [2.00, 4.85]	4.75 [3.00, 6.07]	**<0.001°**
*Major pathological response (%)*	***n*** = **96 (%)**		
MPR	33 (34.4)		
No MPR	63 (65.6)		
*(y)pT (%)*	***n*** = **96 (%)**	***n*** = **114 (%)**	**0.027°**
(y)pT0	1 (1.0)		
(y)pT1	32 (33.3)	17 (14.9)	
(y)pT2	22 (22.9)	37 (32.5)	
(y)pT3	20 (20.8)	31 (27.2)	
(y)pT4	21 (21.9)	29 (25.4)	
*Stage (%)*	***n*** = **96 (%)**	***n*** = **114 (%)**	
I	17 (17.7)		
II	25 (26.0)		
III	50 (52.1)	104 (91.2)	
IV	4 (4.2)	10 (8.8)	
*Growth pattern (%)*		***n*** = **58 (%)**	
Acinar/papillary		15 (25.9)	
Solid		28 (48.3)	
Micropapillary		15 (25.9)	
*Type of resection (%)*	***n*** = **96 (%)**	***n*** = **114 (%)**	0.486*
Wedge		3 (2.6)	
Lobectomy	53 (55.2)	62 (54.4)	
Bilobectomy	5 (5.2)	8 (7.0)	
Pneumonectomy	38 (39.6)	41 (36.0)	
*Neoadjuvant therapy (%)*	***n*** = **94 (%)**		
Cisplatin+Docetaxel	54 (57.4)		
Carboplatin+Paclitaxel	5 (5.3)		
Cisplatin+Pemetrexed	13 (13.8)		
Cisplatin+Gemcitabine	8 (8.5)		
Cisplatin+Vinorelbine	5 (5.3)		
Cisplatin+Etoposide	1 (1.1)		
Other	8 (8.5)		
*Neoadjuvant radiotherapy (%)*	***n*** = **90 (%)**		
No	67 (76.0%)		
Yes	23 (24.0%)		
*Adjuvant therapy (%)*	***n*** = **88 (%)**	***n*** = **99 (%)**	<0.001*
No	65 (73.9)	32 (32.3)	
Yes	23 (26.1)	67 (67.7)	

No statistical comparison of stage, growth patterns, residual tumor and neoadjuvant therapy due to inherent differences.

Main variable names are italic and statistically significant *p*-values are bold.

*Fisher’s exact test, °Wilcoxon rank-sum test.

This study was carried out according to the REMARK criteria and approved by the Cantonal Ethics Commission of the Canton of Bern (KEK 2017–00830), which waived the requirement for written informed consent^28^.

### Survival analyses

We restricted the survival analyses to five years after initial diagnosis to account for the multimorbidity of patients. Overall survival (OS) was defined as the period from the beginning of treatment to death of any cause. DFS was defined as the period from the beginning of treatment to clinically reported relapse or death of any cause. The beginning of treatment was defined by the start of neoadjuvant therapy in the neoadjuvant cohort or the date of resection in the surgical cohort and in 2 cases with missing information about the starting date of neoadjuvant therapy. Patients with stage IV disease (*n* = 14), missing survival information (*n* = 7), non-curative resection (*n* = 2) or last follow-up information within 30 days after surgery (*n* = 12) were excluded from survival analyses resulting in 175 patients included (Supplementary fig. S1). Median OS was 35 (95% CI 29 – NA) months and 87 events were observed (Supplementary fig. S2A). Median DFS was 18 (95% CI 15–25) months and 118 events were observed (Supplementary fig. S2B). There was no significant difference of survival between the neoadjuvant cohort and the surgical cohort (Supplementary fig. S2C, D).

### Immunohistochemical staining and scanning

For immunohistochemical staining appropriate tissue blocks were selected after screening all available H&E slides.

PD-L1 staining was effectuated in a closed system using the Ventana PD-L1 (SP263) assay (Roche Diagnostics International AG, Rotkreuz, Switzerland) on the fully automated immunostainer BenchMark ULTRA (Roche Diagnostics International AG) following the manufacturer’s instructions. The sections were pre-processed using CC1 buffer at 100 °C for 64 min, followed by antibody incubation at 37 °C for 16 min and visualization with DAB.

CD8 staining was effectuated using C8/144B (Agilent, Santa Clara, CA, United States) on the fully automated immunostainer BOND III (Leica Biosystems, Muttenz, Switzerland). The sections were pre-processed with ER2 buffer at 100 °C for 20 min, followed by incubation of the diluted antibody (1:200) for 15 min and visualization with DAB.

Selected slides were digitized using the Pannoramic P250 Flash III (3DHistech, Budapest, Hungary) in multiple runs at a resolution of 0.2431 µm/pixel.

The tissue had been obtained during the routine diagnostic workflow and the formalin-fixed paraffin-embedded tissue had been stored at the Institute of Pathology Bern according to the recommendation of the Swiss Society of Pathology^29^. There was no evidence of time-dependent staining bias with similar distributions of PD-L1 or CD8 expression along the period of observation (Supplementary fig. S3A, B).

### PD-L1 assessment

Specimens with at least 100 tumor cells were eligible. PD-L1 expression was assessed by PZ and reviewed on a double-headed microscope together with SB. In cases of discordant assessment consensus was achieved. PD-L1 expression was assessed as the tumor proportional score (TPS), defined by the proportion of PD-L1 positive tumor cells of all tumor cells. PD-L1 positive tumor cells were defined as showing membranous staining of any intensity. TPS was assessed as a continuous parameter in 1% increments up to 10% and 5% increments in cases showing >10% expression. For statistical analyses, cases were assigned to the three clinically relevant bins of TPS < 1%, 1–49% or ≥50%. PD-L1 positive cases were defined by TPS ≥ 1% and strong expressing cases were defined as TPS ≥ 50%.

### Assessment of CD8 + tumor infiltrating lymphocytes and tertiary lymphoid structures

For the assessment of CD8 + TILs, only biopsies of non-lymph nodes or lymph nodes with desmoplastic reaction were eligible. We evaluated CD8 + TILs per mm^2^ applying a semi-automated approach using the open-source software QuPath (Supplementary fig. S4)^30^. First, we manually annotated regions of interest following recommendations of the International Immuno-Oncology Biomarkers Working Group^31,32^. Thus, only TILs within the borders of the invasive front of tumors were evaluated and smaller satellite nodules without desmoplastic reaction were not included in the assessment. In neoadjuvant cases with extensive fibrotic areas, only the stroma adjacent to the tumor nests was included for analyses. Next, cells in the annotated regions were segmented using the threshold-based watershed detection of QuPath followed by the application of a series of object classifiers for exclusion of anthracotic pigments and artefacts before classification and counting of CD8 negative and positive cells (technical manuscript in preparation). The performance of this automated detection and classification was compared in 22 cases using 5000 ×5000 px wide squares against manual counting of one observer (PZ, Supplementary table 1).

Regarding the evaluation of TLS, the digitized H&E sections were used for manual assessment of the number and activity (presence of germinal centers) of TLS in the resection specimens by PZ^33^. In 44 cases, another block than used for PD-L1 or CD8 assessment was evaluated due to the presence of larger areas of adjacent normal lung tissue. All nodular aggregates of lymphocytes in the tumor region and within 7 mm of the tumor border were counted^33^. In cases of densely infiltrated tumoral stroma, only nodular aggregates apparent on low magnification were included.

### Statistics

All analyses were conducted using R software (version 4.0.5, https://cran.r-project.org/) with suitable packages. For comparison of naturally ordered categorical variables or continuous variables, we used the Wilcoxon rank-sum or Kruskal-Wallis test and for comparison of other categorical variables the Fisher’s exact test. Correlation was assessed using the Spearman test. Survival analyses were conducted using the Log-rank test and univariable cox proportional hazard models. Kaplan-Meier plots were used for the representation of survival curves. Multivariable cox proportional hazard models were used for correction for confounders, which were selected based on a significance level of *p* ≤ 0.1. CD8 + TILs density was included as binary variable (low vs. high) in all survival models. It was dichotomized using maximally selected rank statistics based on Log-rank scores as test statistic and the approximation by Hothorn and Lausen for small sample sizes^34^.

## Results

### No upregulation of PD-L1 expression by neoadjuvant therapy

**Fig. 1:**
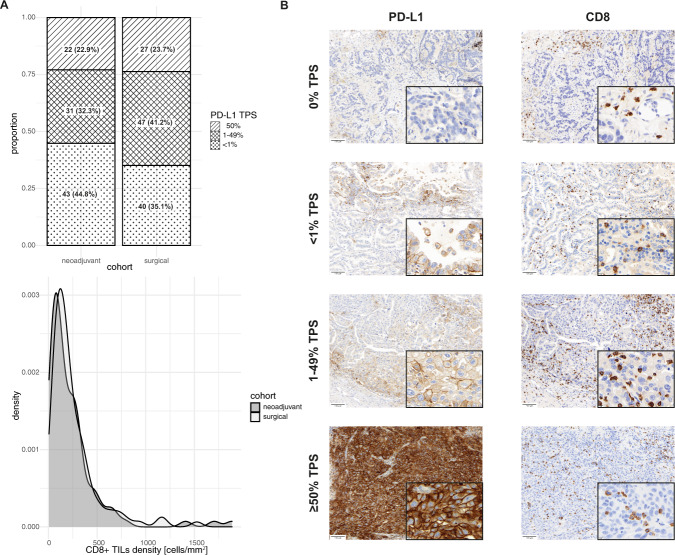
PD-L1 expression and CD8+ TILs. **A** Stacked bar plot and density diagram depict the different distribution of PD-L1 TPS and CD8 + TILs between the neoadjuvant and the surgical cohort. **B** Representative cases of the individual PD-L1 TPS categories and the corresponding CD8 stained section.

After neoadjuvant therapy, PD-L1 expression was <1% in 43/96 (44.8%) cases, 1–49% in 31/96 (32.3%) cases and ≥50% in 22/96 (22.9%) cases (Fig. 1A). In the surgical cohort, PD-L1 expression was <1% in 40/114 (35.1%) cases, 1–49% in 47/114 (41.2) cases and ≥50% in 27/114 (23.7%) cases (Fig. 1A). There was no significant difference in PD-L1 expression between the neoadjuvant cohort and the surgical cohort also after adjusting for histology.

Except for smoking status (active smoker vs. former-/ never smoker) none of the clinico-pathological parameters was associated with higher PD-L1 expression. In the neoadjuvant cohort, active smoking was associated with a higher PD-L1 TPS (*p* = 0.013). Active smokers had a significantly higher frequency of PD-L1 positive tumors in the neoadjuvant cohort (*p* = 0.02, Supplementary fig. S5) and inversely in the surgical cohort (*p* = 0.026, Supplementary fig. S5).

PD-L1 expression was not significantly altered comparing paired pre-/post-neoadjuvant samples. Overall, 7/53 (13.2%) tumors had lower PD-L1 expression and 12/53 (22.6%) had higher PD-L1 expression in the resection specimen, as assessed regarding the clinically significant cut-offs of 1% and 50% (Supplementary table 2 and Supplementary fig. S6). Four/7 cases showed lower PD-L1 expression regarding the 50% cut-off and 5/7 regarding the 1% cut-off (2 cases changed from ≥50% to <1%). A positive or negative change of PD-L1 could not be associated with response to neoadjuvant therapy (major pathological response [MPR] yes/no), patients’ sex, tumor histology or change of CD8 + TILs density (Supplementary table 3).

### Higher CD8 + tumor infiltrating lymphocyte density is associated with higher PD-L1 expression

After neoadjuvant therapy, mean CD8 + TILs density within the tumor region was 242.45 (IQR 73.11–290.32) cells/mm^2^ (Fig. 1A). In the surgical cohort, mean CD8 + TILs density was 252.1 (IQR 98.37–314.73) cells/mm^2^ (Fig. 1A). CD8 + TILs density was similar between the neoadjuvant and surgical cohort. After neoadjuvant therapy, a median of 10 (IQR 4–21.5) TLS were counted on the selected whole slides. In the surgical cohort, a median of 9 (IQR 4.5–19) TLS were counted. After subgrouping according to histology, the median number of TLS was comparable between histological tumor types and groups. However, there were only 4 cases with active TLS in the neoadjuvant cohort compared to 13 cases in the surgical cohort. A higher number of active TLS comparing the cohorts was observed regardless of histological tumor type.

**Fig. 2:**
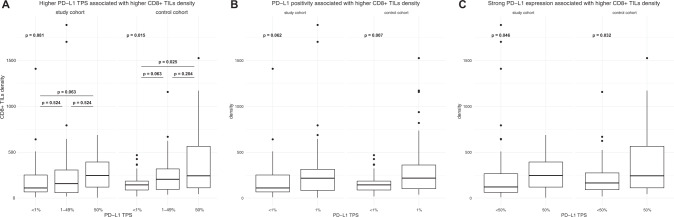
Density of CD8+ TILs according to PD-L1 expression. **A** Distribution of cases using the threefold classification, **B** 1% TPS cut-off and **C** the 50% TPS cut-off.

A higher CD8 + TILs density was statistically significantly associated with LUAD histology (*p*_neoadjuvant_ = 0.001, *p*_surgical_ = 0.017). LUAD showed significantly higher numbers of CD8 + TILs (neoadjuvant: 253.76 [98.42–355.84] vs. 90.43 [51.24–138.39], surgical: 193.49 [128.44–329.61] vs. 137.91 [77.34–242.90]). Additional neoadjuvant radiotherapy (*p*_neoadjuvant_ = 0.026) and a higher PD-L1 expression (*p*_neoadjuvant_ = 0.027 R_S_ = 0.23, *p*_surgical_ = 0.003 R_S_ = 0.28, Fig. 2A) were associated with increased densities of CD8 + TILs. However, when subgrouping according to histological tumor type, PD-L1 expression did no longer significantly correlate with CD8 + TILs density in LUAD after neoadjuvant treatment. Similar results were observed when applying the clinical cut-offs at 1% or 50% PD-L1 expression (Fig. 2B, C). Among the primary resected cases, PD-L1 positive cases showed a higher CD8 + TILs density. After neoadjuvant therapy, this remained true only for non-LUAD tumors. Strong PD-L1 expression correlated with CD8 + TILs density in LUAD and non-LUAD tumors in both cohorts. In the surgical cohort, tumor size inversely correlated with CD8 + TILs density (R_s_ = −0.24, *p* = 0.011), whereas in cases after neoadjuvant treatment, higher numbers of TLS correlated with higher CD8 + TILs density (R_s_ = 0.27, *p* = 0.009).

**Fig. 3:**
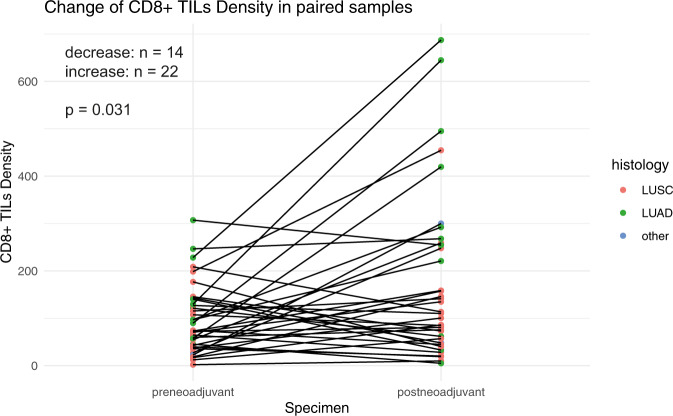
Change of CD8 + TILs density in paired specimens, comparing diagnostic biopsies vs. resection specimens after neoadjuvant therapy. The histological tumor types visualized by different colors indicate a potentially more pronounced positive change in lung adenocarcinomas compared to tumors with non-adenocarcinoma histology. LUSC lung squamous cell carcinoma, LUAD lung adenocarcinoma.

CD8 + TILs density was significantly lower before neoadjuvant therapy comparing paired samples (*p* = 0.031, Fig. 3). We performed subgroup analyses to check whether changes of CD8 + TILs density were associated with changes in PD-L1 expression. However, changes of CD8 + TILs density were only significant in the subgroup of cases with no change of PD-L1 expression regarding the three-fold classification (Supplementary Fig. S7), presumably due to insufficient sample size in the other subgroups. Furthermore, higher CD8 + density before or after neoadjuvant therapy was not associated with an increase of PD-L1 expression.

### Prognostic significance of immune related biomarkers

**Fig. 4:**
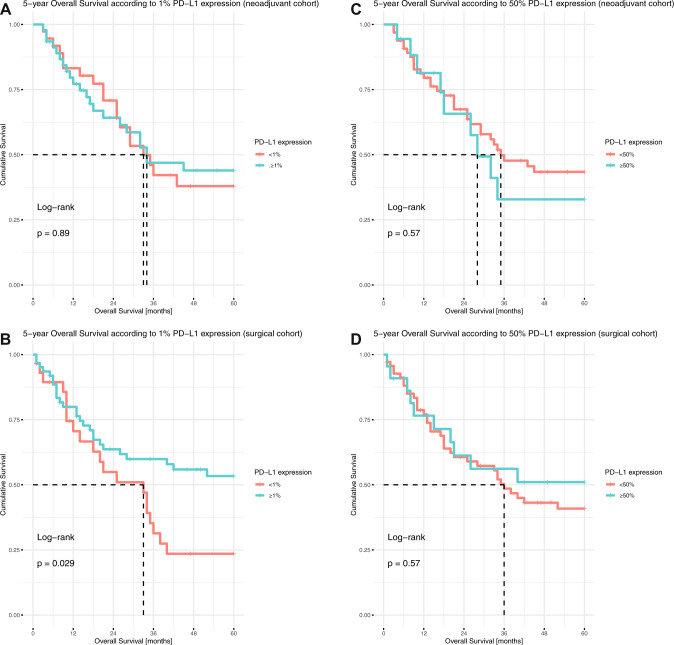
Evaluating the prognostic value of PD-L1 expression. **A**, **C** Kaplan–Meier plots of the neoadjuvant cohort and **B**, **D** the surgical cohort according to the **A**, **B** 1% TPS cut-off and **C**, **D** 50% TPS cut-off.

**Fig. 5:**
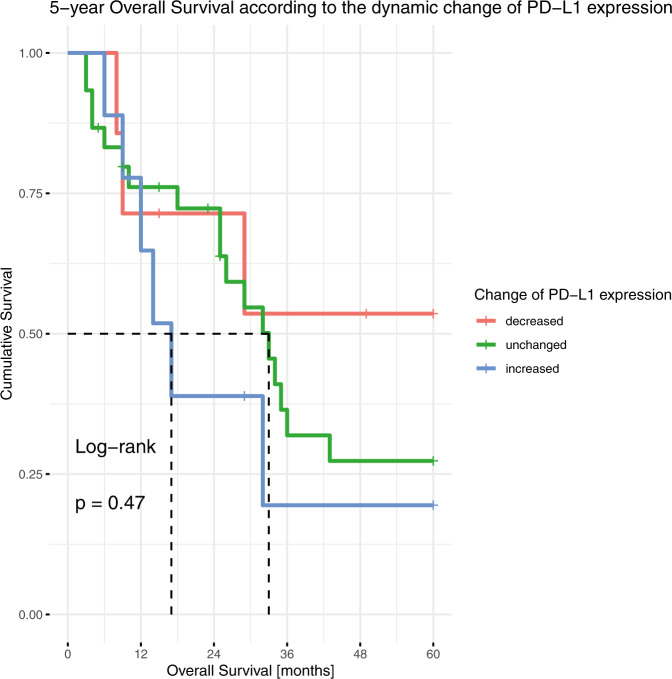
Evaluating the prognostic value of an altered PD-L1 expression after neoadjuvant chemotherapy. Kaplan-Meier plot of the dynamics of PD-L1 expression change (based on the three-tier classification with cut-offs 1% and 50%).

In the entire study population, PD-L1 expression assessed in resection specimens had no prognostic significance, neither for OS nor for DFS, neither using the three-tier classification nor the cut-offs of 1% or 50% individually (Supplementary fig. S8). In subgroup analyses including only cases after neoadjuvant therapy or primary resected cases, PD-L1 positivity was a prognostic marker for longer OS in the surgical cohort (*p* = 0.029, HR 0.5255, 95% CI 0.2924–0.9444, Fig. 4). Regarding PD-L1 as a dynamic marker, although patients with decreased PD-L1 expression seemed to have a longer 5-year OS, this was not statistically significant, and the case number was very low (Fig. 5).

**Fig. 6:**
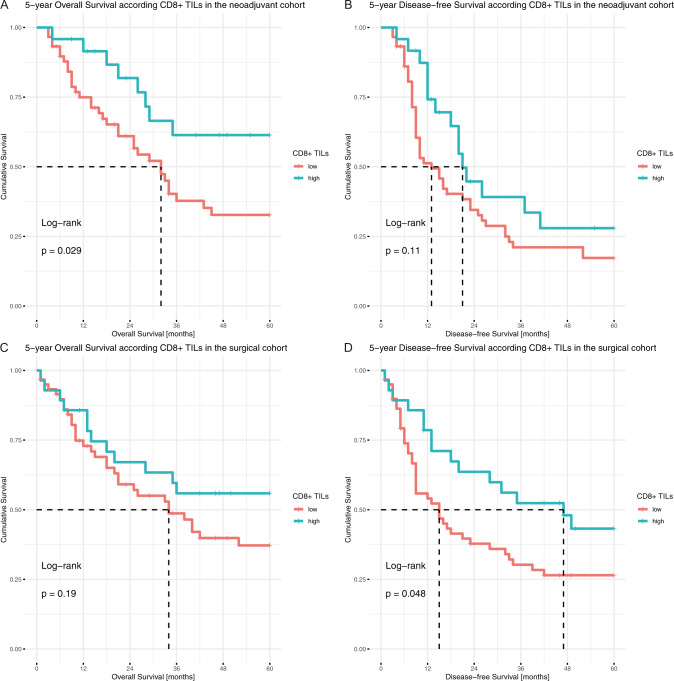
Evaluating the prognostic value of CD8+ TILs density. Kaplan-Meier plots of CD8 + TILs density regarding **A**, **C** overall survival and **B**, **D** disease-free survival in **A**, **B** the neoadjuvant cohort and **C**, **D** the surgical cohort.

We used maximally selected rank statistic to determine the adequate cut-off for CD8 + TILs density at 283.18 cells/mm^2^ in the entire study population. Overall, higher CD8 + TILs numbers were associated with longer OS (*p* = 0.014, HR 0.5373, 95% CI 0.3251–0.888, Supplementary fig. S9A) and longer DFS (*p* = 0.008, HR 0.5707, 95% CI 0.3762–0.8656, Supplementary fig. S9B). In the subgroup analyses, however, it was a positive prognostic factor for OS only in the neoadjuvant cohort (*p* = 0.029, HR 0.4332, 95% CI 0.1997–0.9397, Fig. 6A) and for DFS only in the surgical cohort (*p* = 0.048, HR 0.5513, 95% CI 0.3043–0.9986, Fig. 6D).

The number of TLS was considered as a continuous variable and it was a prognostic marker considering the entire study population (*p* = 0.045, HR 0.9833, 95% CI 0.9673–0.9996) but not in the sub-cohorts (*p*_neoadjuvant_ = 0.15, *p*_surgical_ = 0.15).

We investigated the validity of the prognostic significance only in the overall population to achieve sufficient sample size and number of events. As the three immune markers PD-L1 expression, CD8 + TILs and TLS correlated significantly, we performed a cox regression analysis for each marker. PD-L1 expression was included using the TPS 1% cut-off only. None of the immune markers was prognostic in the multivariable model for OS (p_PD-L1_ = 0.241, p_CD8+_ = 0.368, p_TLS_ = 0.246) or DFS (p_PD-L1_ = 0.054, p_CD8+_ = 0.098, p_TLS_ = 0.407) but age and pT4 were consistent prognostic factors in the multivariable models (Supplementary fig. S10).

## Discussion

In this study on immune related biomarkers in locally advanced resectable NSCLC, CD8 + TILs and TLS were prognostic factors but did not yield additional information to age and TNM in multivariable analyses. CD8 + TILs density correlated with PD-L1 expression. PD-L1 expression was not consistently upregulated after neoadjuvant chemotherapy, in line with some, but not all previous studies^13,16,19–22^.

The effect of chemotherapy on PD-L1 expression in the resectable setting has been previously investigated as summarized in Table 1^11–23^. Most studies reported an increased PD-L1 expression after chemotherapy when assessing paired samples. The importance of the PI3K/ALK pathway in PD-L1 upregulation after chemotherapy was demonstrated in in vitro and in vivo functional assays^14,19^. Conversely, some studies showed no change or even a decreased PD-L1 expression following chemotherapy^11,12,15,17,18^. In fact, when reassessing the positive studies and considering the absolute number of cases per category (*decrease, no change, increase*), most cases did not change. Of the four studies conducted using an FDA approved antibody, only one concluded an increased PD-L1 expression post-chemotherapy^15,17,18,20^. Thus, our study results are in line with previous reports for FDA approved antibodies and suggest that changes in PD-L1 expression due to chemotherapy are observed only in a minority of tumors. In the majority of our cases (34/53) PD-L1 expression was stable and most of them were negative (21/34 cases) in both the biopsy/cytology specimens and the resection after neoadjuvant therapy. This concordance between biopsy and resection in explicit cases (TPS < 1% or ≥50%) was also described in a larger study by Hwang et al. performed on 103 paired cases without focus on neoadjuvant therapy^35^. The authors reported only a moderate concordance, primarily explained by considerable discordance in cases with intermediate PD-L1 expression (TPS 1–49%)^35^. Considering these and previously reported results, it seems unlikely that neoadjuvant chemotherapy induces PD-L1 expression.

Chemotherapy is deemed to improve immunosurveillance by different effects (antigenicity, immunogenicity, susceptibility)^36^. This would suggest an upregulation of PD-L1 in tumor cells to evade a strong antitumor response. A potential explanation for the lack of consistent upregulation in clinical samples could be the influence of chemotherapy on the immune microenvironment. Two larger studies have investigated such effects in detail by applying multiplex immunofluorescence and a multiomics approach^16,23^. Both studies describe an increase of immune cells in specimens after neoadjuvant therapy and point out that different subgroups of immune cells are increased depending on the underlying histological tumor type^16,23^. However, the multiomics approach indicated no increase of T-cell receptor richness and clonality, thus failed to validate the supposed increase of antigenicity or susceptibility^23^. Importantly, these studies did not include paired samples and, especially the study using multiomics, included only few cases depending on the performed analysis (*n* = 10–72)^23^.

In order to account for the immune microenvironment, we assessed the CD8 + TILs density and number and state of TLS. By applying a semiautomatic approach, we were able to count CD8 + TILs in the entire tumor region of the slides used for PD-L1 assessment, in contrast to the published studies usually performing hot spot analysis^37–40^. Although we confirmed published results indicating an increase of TILs in tumors resected after neoadjuvant therapy^15,18,22^, we cannot conclude that chemotherapy increases CD8 + TILs densities due to (a) no significant difference in CD8 + density between cases resected after neoadjuvant therapy and primary resected cases, (b) biopsies covering much smaller tissue areas than whole slides (thus being more prone to sampling error due to heterogeneity), and (c) higher variance in post-neoadjuvant therapy specimens leading to higher median ranks and means due to outliers (implications for statistical testing).

Another explanation, comparing preclinical and clinical studies, could be the heterogeneity of applied chemotherapeutic regimens, inherent in our real-life cohort approach. Although most cytotoxic agents have immunosurveillance enhancing effects, these differ considerably^36^. Thus, while oxaliplatin and gemcitabine have been shown to promote immunogenic cell death, especially via exposure of calreticulin, others do not without addition of radiotherapy (e.g. cisplatin)^36,41^. Furthermore, although Zhang et al. and Fournel et al. suggested an upregulation of PD-L1 via the PI3K/ALK pathway, earlier studies support rather the downregulation of suppressive checkpoints via the STAT pathway^14,19,41^. In our study, the majority of patients (*n* = 54) received cisplatin and docetaxel, but only 12/54 received additional radiotherapy^41^. On the other hand, the few patients who had received gemcitabine and had available paired samples (*n* = 2) showed PD-L1-upregulation despite the lack of neoadjuvant radiotherapy, and only 3/11 cases were PD-L1 negative in the resection specimens.

The prognostic power of PD-L1 expression as a double-edged sword has already been described exhaustively in advanced NSCLC and a high PD-L1 expression does not seem to be consistently associated with shorter survival^42^. Likewise, most studies including tumors after neoadjuvant therapy reported no prognostic importance of PD-L1 expression^11,15,18,20–22^. In this study, static PD-L1 expression, evaluated only in the resection specimens, was not prognostic. However, the dynamic changes resulting in increased PD-L1 expression could confer worse survival, in accordance with previous studies^12,14,15,21^. Presumably due to the low sample size, this could not be statistically confirmed in our cohort. This effect could be explained by PD-L1 expression potentially conveying chemoresistance and promoting proliferation and epithelial to mesenchymal transition^14,43^. In the literature and in our study population (combined neoadjuvant and surgical cohort), high CD8 + TILs were commonly associated with improved OS^11,14,18,22,44,45^. This seems to be true even when applying different cut-offs, as most of the published studies used the median, thus a cohort specific cut-off^11,15,18,22,46^. Furthermore, the prognostic impact of a higher CD8 + TILs density is a possible explanation for the prognostic benefit of PD-L1 positivity in primary LUSC, due to the positive correlation of PD-L1 expression and CD8 + TILs. In our cohort, however, CD8 + TILs lost its prognostic relevance in multivariable analyses including age and the pT denominator of the TNM classification. Thus, we cannot confirm the importance of the immune pattern as a complementary factor for survival prediction, as proposed by Remark and colleagues^11^.

This retrospective study assessed the impact of chemotherapy on biomarkers for ICB in a real-life cohort resected after neoadjuvant therapy over a period of 16 years. The availability of paired samples for 53 patients is comparable to prior studies but the addition of a matching cohort of primary resected locally advanced NSCLC allowed the validation of identified effects. In contrast to most published studies, we evaluated PD-L1 expression according to the current diagnostic recommendations and using an FDA-approved antibody assay for companion diagnostics^47–49^. Furthermore, we included the impact on the immune microenvironment by evaluating CD8 + TILs and TLS. Contrary to published studies, our approach to evaluate TILs was based on an open-source software, was independent from the histological tumor type and addressed lung-specific artefacts as anthracosis, allowing a reliable assessment of CD8 + TILs on the whole slide level corresponding to the area assessed for PD-L1 scoring^37–40,50^.

Nevertheless, our study has limitations inherent to its retrospective and “real-life” character. In particular, compared to clinical studies investigating the effect of (radio-)chemotherapy, patients with different chemotherapeutic regimens were included^15,18^. Although patients with changed PD-L1 expression after neoadjuvant therapy did not statistically differ from the rest of the cohort regarding duration of neoadjuvant therapy or therapy free interval between the last cycle of neoadjuvant therapy and resection, these differences in duration of therapy and therapy free interval need to be accounted for when interpreting our results.

In conclusion, PD-L1 expression was not altered by intervening neoadjuvant (radio-)chemotherapy but CD8 + TILs were significantly increased. However, cases with intermediate PD-L1 expression (1–49% TPS) in biopsies showed some dynamic (6/12 [50%] cases changed PD-L1 expression in the resection) compared to negative (<1% PD-L1 TPS) or strong positive (≥50% PD-L1 TPS) cases (approximately 30% changed PD-L1 expression in the resection). Thus, it could be of value to repeat PD-L1 testing in cases with intermediate PD-L1 expression. Literature suggests PD-L1 dynamics (*decrease*, *no change*, *increase*) to be a more valid prognostic marker. Although maybe due to the low sample size, this could not be statistically confirmed in our cohort. Subsequent clinical trials are warranted in order to determine if PD-L1 retesting should be performed after neoadjuvant therapy due to therapeutic implications of an altered PD-L1 expression.

## Supplementary information

supplemental Material

## Data Availability

Anonymized detailed clinico-pathological data and the R-Script used for data analysis are available upon request to the authors.

## References

[1] Sung, H., Ferlay, J., Siegel, R. L., Laversanne, M., Soerjomataram, I., Jemal, A. et al. Global cancer statistics 2020: GLOBOCAN estimates of incidence and mortality worldwide for 36 cancers in 185 countries. *CA Cancer J Clin* **71**, 209–249 (2021). 10.3322/caac.21660 33538338

[2] Howington, J. A., Blum, M. G., Chang, A. C., Balekian, A. A. & Murthy, S. C. Treatment of stage I and II non-small cell lung cancer: Diagnosis and management of lung cancer, 3rd ed: American College of Chest Physicians evidence-based clinical practice guidelines. *Chest* **143**, e278S–e313S (2013). 10.1378/chest.12-2359 23649443

[3] Souquet, P. J. & Geriniere, L. The role of chemotherapy in early stage of non-small cell lung cancer. *Lung Cancer* **34**, S155–158 (2001). 10.1016/s0169-5002(01)00361-0 11720758

[4] Postmus, P. E., Kerr, K. M., Oudkerk, M., Senan, S., Waller, D. A., Vansteenkiste, J. et al. Early and locally advanced non-small-cell lung cancer (NSCLC): ESMO Clinical Practice Guidelines for diagnosis, treatment and follow-up. *Ann Oncol* **28**, iv1-iv21 (2017). 10.1093/annonc/mdx222 28881918

[5] Pignon, J. P., Tribodet, H., Scagliotti, G. V., Douillard, J. Y., Shepherd, F. A., Stephens, R. J. et al. Lung adjuvant cisplatin evaluation: a pooled analysis by the LACE Collaborative Group. *J Clin Oncol* **26**, 3552–3559 (2008). 10.1200/JCO.2007.13.9030 18506026

[6] Spicer, J., Wang, C., Tanaka, F., Saylors, G. B., Chen, K.-N., Liberman, M. et al. Surgical outcomes from the phase 3 CheckMate 816 trial: Nivolumab (NIVO) + platinum-doublet chemotherapy (chemo) vs chemo alone as neoadjuvant treatment for patients with resectable non-small cell lung cancer (NSCLC). *Journal of Clinical Oncology* **39**, 8503–8503 (2021).

[7] Wakelee, H. A., Altorki, N. K., Zhou, C., Csőszi, T., Vynnychenko, I. O., Goloborodko, O. et al. IMpower010: Primary results of a phase III global study of atezolizumab versus best supportive care after adjuvant chemotherapy in resected stage IB-IIIA non-small cell lung cancer (NSCLC). *Journal of Clinical Oncology* **39**, 8500–8500 (2021).

[8] Wei, S. C., Duffy, C. R. & Allison, J. P. Fundamental Mechanisms of Immune Checkpoint Blockade Therapy. *Cancer Discov* **8**, 1069–1086 (2018). 10.1158/2159-8290.CD-18-0367 30115704

[9] Dafni, U., Tsourti, Z., Vervita, K. & Peters, S. Immune checkpoint inhibitors, alone or in combination with chemotherapy, as first-line treatment for advanced non-small cell lung cancer. A systematic review and network meta-analysis. *Lung Cancer* **134**, 127–140 (2019). 10.1016/j.lungcan.2019.05.029 31319971

[10] Camidge, D. R., Doebele, R. C. & Kerr, K. M. Comparing and contrasting predictive biomarkers for immunotherapy and targeted therapy of NSCLC. *Nat Rev Clin Oncol* **16**, 341–355 (2019). 10.1038/s41571-019-0173-9 30718843

[11] Remark, R., Lupo, A., Alifano, M., Biton, J., Ouakrim, H., Stefani, A. et al. Immune contexture and histological response after neoadjuvant chemotherapy predict clinical outcome of lung cancer patients. *Oncoimmunology* **5**, e1255394 (2016). 10.1080/2162402X.2016.1255394 PMC5213838 28123901

[12] Sheng, J., Fang, W., Yu, J., Chen, N., Zhan, J., Ma, Y. et al. Expression of programmed death ligand-1 on tumor cells varies pre and post chemotherapy in non-small cell lung cancer. *Sci Rep* **6**, 20090 (2016). 10.1038/srep20090 PMC4731819 26822379

[13] Song, Z., Yu, X. & Zhang, Y. Altered expression of programmed death-ligand 1 after neo-adjuvant chemotherapy in patients with lung squamous cell carcinoma. *Lung Cancer* **99**, 166–171 (2016). 10.1016/j.lungcan.2016.07.013 27565935

[14] Zhang, P., Ma, Y., Lv, C., Huang, M., Li, M., Dong, B. *et al*. Upregulation of programmed cell death ligand 1 promotes resistance response in non-small-cell lung cancer patients treated with neo-adjuvant chemotherapy. *Cancer Sci* **107**, 1563–1571 (2016). 10.1111/cas.13072 PMC5132280 27581532

[15] Fujimoto, D., Uehara, K., Sato, Y., Sakanoue, I., Ito, M., Teraoka, S. et al. Alteration of PD-L1 expression and its prognostic impact after concurrent chemoradiation therapy in non-small cell lung cancer patients. *Sci Rep* **7**, 11373 (2017). 10.1038/s41598-017-11949-9 PMC5595796 28900290

[16] Parra, E. R., Villalobos, P., Behrens, C., Jiang, M., Pataer, A., Swisher, S. G. et al. Effect of neoadjuvant chemotherapy on the immune microenvironment in non-small cell lung carcinomas as determined by multiplex immunofluorescence and image analysis approaches. *J Immunother Cancer* **6**, 48 (2018). 10.1186/s40425-018-0368-0 PMC5989476 29871672

[17] Rojko, L., Reiniger, L., Teglasi, V., Fabian, K., Pipek, O., Vagvolgyi, A. et al. Chemotherapy treatment is associated with altered PD-L1 expression in lung cancer patients. *J Cancer Res Clin Oncol* **144**, 1219–1226 (2018). 10.1007/s00432-018-2642-4 PMC11813485 29675791

[18] Choe, E. A., Cha, Y. J., Kim, J. H., Pyo, K. H., Hong, M. H., Park, S. Y. et al. Dynamic changes in PD-L1 expression and CD8(+) T cell infiltration in non-small cell lung cancer following chemoradiation therapy. *Lung Cancer* **136**, 30–36 (2019). 10.1016/j.lungcan.2019.07.027 31421259

[19] Fournel, L., Wu, Z., Stadler, N., Damotte, D., Lococo, F., Boulle, G. et al. Cisplatin increases PD-L1 expression and optimizes immune check-point blockade in non-small cell lung cancer. *Cancer Lett* **464**, 5–14 (2019). 10.1016/j.canlet.2019.08.005 31404614

[20] Guo, L., Song, P., Xue, X., Guo, C., Han, L., Fang, Q. et al. Variation of Programmed Death Ligand 1 Expression After Platinum-based Neoadjuvant Chemotherapy in Lung Cancer. *J Immunother* **42**, 215–220 (2019). 10.1097/CJI.0000000000000275 PMC6587215 31145232

[21] Shin, J., Chung, J. H., Kim, S. H., Lee, K. S., Suh, K. J., Lee, J. Y. et al. Effect of Platinum-Based Chemotherapy on PD-L1 Expression on Tumor Cells in Non-small Cell Lung Cancer. *Cancer Res Treat* **51**, 1086–1097 (2019). 10.4143/crt.2018.537 PMC6639222 30428640

[22] Yoneda, K., Kuwata, T., Kanayama, M., Mori, M., Kawanami, T., Yatera, K. et al. Alteration in tumoural PD-L1 expression and stromal CD8-positive tumour-infiltrating lymphocytes after concurrent chemo-radiotherapy for non-small cell lung cancer. *Br J Cancer* **121**, 490–496 (2019). 10.1038/s41416-019-0541-3 PMC6738061 31388183

[23] Gaudreau, P. O., Negrao, M. V., Mitchell, K. G., Reuben, A., Corsini, E. M., Li, J. et al. Neoadjuvant Chemotherapy Increases Cytotoxic T Cell, Tissue Resident Memory T Cell, and B Cell Infiltration in Resectable NSCLC. *J Thorac Oncol* **16**, 127–139 (2021). 10.1016/j.jtho.2020.09.027 PMC7775914 33096269

[24] Pfirschke, C., Engblom, C., Rickelt, S., Cortez-Retamozo, V., Garris, C., Pucci, F. et al. Immunogenic Chemotherapy Sensitizes Tumors to Checkpoint Blockade Therapy. *Immunity* **44**, 343–354 (2016). 10.1016/j.immuni.2015.11.024 PMC4758865 26872698

[25] Zens, P., Bello, C., Scherz, A., Koenigsdorf, J., Pollinger, A., Schmid, R. A. et al. A prognostic score for non-small cell lung cancer resected after neoadjuvant therapy in comparison with the tumor-node-metastases classification and major pathological response. *Mod Pathol* **34**, 1333–1344 (2021). 10.1038/s41379-021-00777-y PMC8216907 33714982

[26] Brierley JD, G. M., Wittekind C. *International Union Against Cancer (UICC): TNM Classification of Malignant Tumours*. 8 edn. (Wiley-Blackwell, 2017).

[27] WHO Classification of Tumours. *Thoracic Tumours*. 5 edn, Vol. 5 (International Agency for Research on Cancer, 2021).

[28] McShane, L. M., Altman, D. G., Sauerbrei, W., Taube, S. E., Gion, M., Clark, G. M. et al. Reporting recommendations for tumor marker prognostic studies (REMARK). *J Natl Cancer Inst* **97**, 1180–1184 (2005). 10.1093/jnci/dji237 16106022

[29] Dirnhofer, S., Bubendorf, L., Lehr, H.-A., Landau, B. & Zenklusen, H.-R. Quality guidelines of the Swiss Society of Pathology. Qualitätsrichtlinien SGPath (St. Gallen, Schweizerische Gesellschaft für Pathologie, accessed 29 July 2022); https://sgpath.ch/qualitaetssicherung/ (2011).

[30] Bankhead, P., Loughrey, M. B., Fernandez, J. A., Dombrowski, Y., McArt, D. G., Dunne, P. D. et al. QuPath: Open source software for digital pathology image analysis. *Sci Rep* **7**, 16878 (2017). 10.1038/s41598-017-17204-5 PMC5715110 29203879

[31] Hendry, S., Salgado, R., Gevaert, T., Russell, P. A., John, T., Thapa, B. et al. Assessing Tumor-infiltrating Lymphocytes in Solid Tumors: A Practical Review for Pathologists and Proposal for a Standardized Method From the International Immunooncology Biomarkers Working Group: Part 1: Assessing the Host Immune Response, TILs in Invasive Breast Carcinoma and Ductal Carcinoma In Situ, Metastatic Tumor Deposits and Areas for Further Research. *Adv Anat Pathol* **24**, 235–251 (2017). 10.1097/PAP.0000000000000162 PMC5564448 28777142

[32] Hendry, S., Salgado, R., Gevaert, T., Russell, P. A., John, T., Thapa, B. et al. Assessing Tumor-Infiltrating Lymphocytes in Solid Tumors: A Practical Review for Pathologists and Proposal for a Standardized Method from the International Immuno-Oncology Biomarkers Working Group: Part 2: TILs in Melanoma, Gastrointestinal Tract Carcinomas, Non-Small Cell Lung Carcinoma and Mesothelioma, Endometrial and Ovarian Carcinomas, Squamous Cell Carcinoma of the Head and Neck, Genitourinary Carcinomas, and Primary Brain Tumors. *Adv Anat Pathol* **24**, 311–335 (2017). 10.1097/PAP.0000000000000161 PMC5638696 28777143

[33] Silina, K., Soltermann, A., Attar, F. M., Casanova, R., Uckeley, Z. M., Thut, H. et al. Germinal Centers Determine the Prognostic Relevance of Tertiary Lymphoid Structures and Are Impaired by Corticosteroids in Lung Squamous Cell Carcinoma. *Cancer Res* **78**, 1308–1320 (2018). 10.1158/0008-5472.CAN-17-1987 29279354

[34] Hothorn, T. & Lausen, B. On the exact distribution of maximally selected rank statistics. *Computational Statistics & Data Analysis* **43**, 121–137 (2003).

[35] Hwang, D. M., Albaqer, T., Santiago, R. C., Weiss, J., Tanguay, J., Cabanero, M. et al. Prevalence and Heterogeneity of PD-L1 Expression by 22C3 Assay in Routine Population-Based and Reflexive Clinical Testing in Lung Cancer. *J Thorac Oncol* **16**, 1490–1500 (2021). 10.1016/j.jtho.2021.03.028 33915250

[36] Zitvogel, L., Galluzzi, L., Smyth, M. J. & Kroemer, G. Mechanism of action of conventional and targeted anticancer therapies: reinstating immunosurveillance. *Immunity* **39**, 74–88 (2013). 10.1016/j.immuni.2013.06.014 23890065

[37] Parra, E. R., Behrens, C., Rodriguez-Canales, J., Lin, H., Mino, B., Blando, J. et al. Image Analysis-based Assessment of PD-L1 and Tumor-Associated Immune Cells Density Supports Distinct Intratumoral Microenvironment Groups in Non-small Cell Lung Carcinoma Patients. *Clin Cancer Res* **22**, 6278–6289 (2016). 10.1158/1078-0432.CCR-15-2443 PMC5558040 27252415

[38] Kim, M. Y., Koh, J., Kim, S., Go, H., Jeon, Y. K. & Chung, D. H. Clinicopathological analysis of PD-L1 and PD-L2 expression in pulmonary squamous cell carcinoma: Comparison with tumor-infiltrating T cells and the status of oncogenic drivers. *Lung Cancer* **88**, 24–33 (2015). 10.1016/j.lungcan.2015.01.016 25662388

[39] Koh, J., Go, H., Keam, B., Kim, M. Y., Nam, S. J., Kim, T. M. et al. Clinicopathologic analysis of programmed cell death-1 and programmed cell death-ligand 1 and 2 expressions in pulmonary adenocarcinoma: comparison with histology and driver oncogenic alteration status. *Mod Pathol* **28**, 1154–1166 (2015). 10.1038/modpathol.2015.63 26183759

[40] Conde, E., Caminoa, A., Dominguez, C., Calles, A., Walter, S., Angulo, B. et al. Aligning digital CD8(+) scoring and targeted next-generation sequencing with programmed death ligand 1 expression: a pragmatic approach in early-stage squamous cell lung carcinoma. *Histopathology* **72**, 270–284 (2018). 10.1111/his.13346 28815764

[41] Tsao, M. S., Kerr, K. M., Dacic, S., Yatabe, Y. & Hirsch, F. R. IASLC Atlas of PD-L1 Immunohistochemistry Testing in Lung Cancer (North Fort Myers, Editorial Rx Press, 2017).

[42] Takada, K., Toyokawa, G., Shoji, F., Okamoto, T. & Maehara, Y. The Significance of the PD-L1 Expression in Non-Small-Cell Lung Cancer: Trenchant Double Swords as Predictive and Prognostic Markers. *Clin Lung Cancer* **19**, 120–129 (2018). 10.1016/j.cllc.2017.10.014 29153898

[43] Shimoji, M., Shimizu, S., Sato, K., Suda, K., Kobayashi, Y., Tomizawa, K. et al. Clinical and pathologic features of lung cancer expressing programmed cell death ligand 1 (PD-L1). *Lung Cancer* **98**, 69–75 (2016). 10.1016/j.lungcan.2016.04.021 27393509

[44] Bremnes, R. M., Busund, L. T., Kilvaer, T. L., Andersen, S., Richardsen, E., Paulsen, E. E. et al. The Role of Tumor-Infiltrating Lymphocytes in Development, Progression, and Prognosis of Non-Small Cell Lung Cancer. *J Thorac Oncol* **11**, 789–800 (2016). 10.1016/j.jtho.2016.01.015 26845192

[45] Fumet, J. D., Richard, C., Ledys, F., Klopfenstein, Q., Joubert, P., Routy, B. et al. Prognostic and predictive role of CD8 and PD-L1 determination in lung tumor tissue of patients under anti-PD-1 therapy. *Br J Cancer* **119**, 950–960 (2018). 10.1038/s41416-018-0220-9 PMC6203820 30318514

[46] Chen, L., Cao, M. F., Zhang, X., Dang, W. Q., Xiao, J. F., Liu, Q. et al. The landscape of immune microenvironment in lung adenocarcinoma and squamous cell carcinoma based on PD-L1 expression and tumor-infiltrating lymphocytes. *Cancer Med* **8**, 7207–7218 (2019). 10.1002/cam4.2580 PMC6885882 31605439

[47] Tsao, M. S., Kerr, K. M., Dacic, S., Yatabe, Y. & Hirsch, F. R. IASLC Atals of PD-L1 immunohistochemistry testing in lung cancer. (North Fort Myers: Editorial Rx Press, 2017).

[48] Naito, T., Udagawa, H., Sato, J., Horinouchi, H., Murakami, S., Goto, Y. et al. A Minimum Of 100 Tumor Cells in a Single Biopsy Sample Is Required to Assess Programmed Cell Death Ligand 1 Expression in Predicting Patient Response to Nivolumab Treatment in Nonsquamous Non-Small Cell Lung Carcinoma. *J Thorac Oncol* **14**, 1818–1827 (2019). 10.1016/j.jtho.2019.06.019 31260834

[49] Twomey, J. D. & Zhang, B. Cancer Immunotherapy Update: FDA-Approved Checkpoint Inhibitors and Companion Diagnostics. *AAPS J* **23**, 39 (2021). 10.1208/s12248-021-00574-0 PMC7937597 33677681

[50] Kilvaer, T. K., Paulsen, E. E., Andersen, S., Rakaee, M., Bremnes, R. M., Busund, L. R. et al. Digitally quantified CD8+ cells: the best candidate marker for an immune cell score in non-small cell lung cancer? *Carcinogenesis* **41**, 1671–1681 (2020). 10.1093/carcin/bgaa105 PMC7791621 33035322

