# Supplementary material for: The effect of neoadjuvant therapy on PD-L1 expression and CD8+lymphocyte density in non-small cell lung cancer
Source: Mod Pathol. 2022 Aug 1;35(12):1848–59. doi: 10.1038/s41379-022-01139-y (PMC9708547; doi:10.1038/s41379-022-01139-y)
Supplement: Supplementary file 1 — supplemental Material [file 41379_2022_1139_MOESM1_ESM.docx]

# Supplementary Material

| **Element** | **Title** | **Page** |
| --- | --- | --- |
| Figure S1 | Case selection for the different analyses | 2 |
| Figure S2 | Overall and disease-free survival of the study population | 3 |
| Figure S3 | Time-dependent change of marker expression | 4 |
| Figure S4 | Semi-automated lymphocyte detection | 5 |
| Figure S5 | Association of smoking status with PD-L1 TPS | 6 |
| Figure S6 | Change of raw PD-L1 TPS according to specimen | 7 |
| Figure S7 | CD8 TILs according to PD-L1 change (based on three tier classification of PD-L1) | 9 |
| Figure S8 | Prognostic significance of PD-L1 in locally advanced resectable NSCLC | 10 |
| Figure S9 | CD8+ TILs are a prognostic factor in locally-advanced NSCLC | 11 |
| Figure S10 | Multivariable cox proportional hazard models for prognostic markers | 12 |
| Table S1 | Comparison of manual and automatic TILs detection | 13 |
| Table S2 | Cases with changed PD-L1 expression after neoadjuvant therapy | 14 |
| Table S3 | Association of change of PD-L1 expression with clinico-pathological parameters | 15 |

# Supplementary figure S1: Case selection for the different analyses


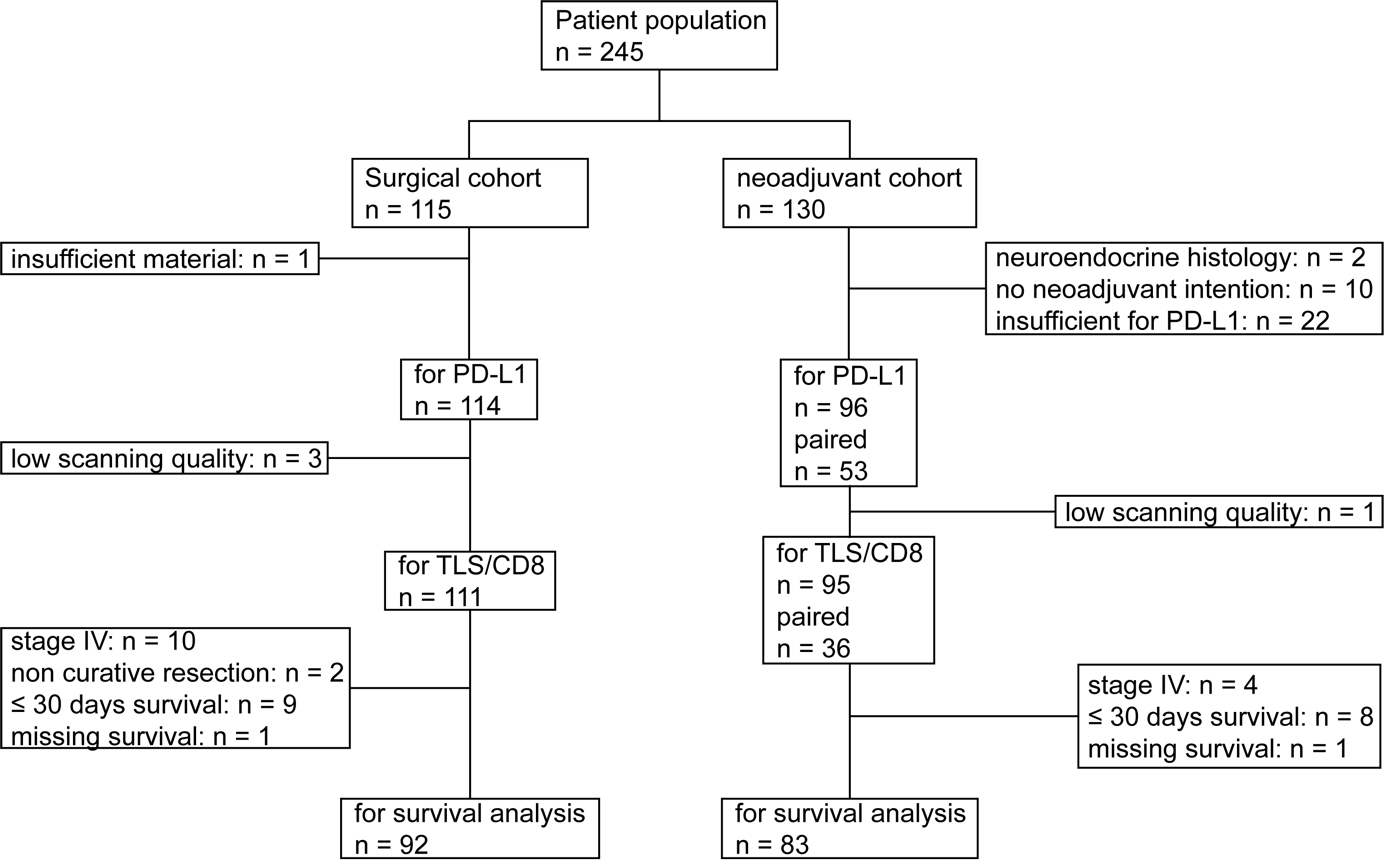


Figure S1: Selection of patients in the neoadjuvant and surgical cohorts. All patients in whom PD-L1 was assessed were eligible for survival analysis if not excluded due to the specified reasons.

# Supplementary figure S2: Overall and disease-free survival of the study population


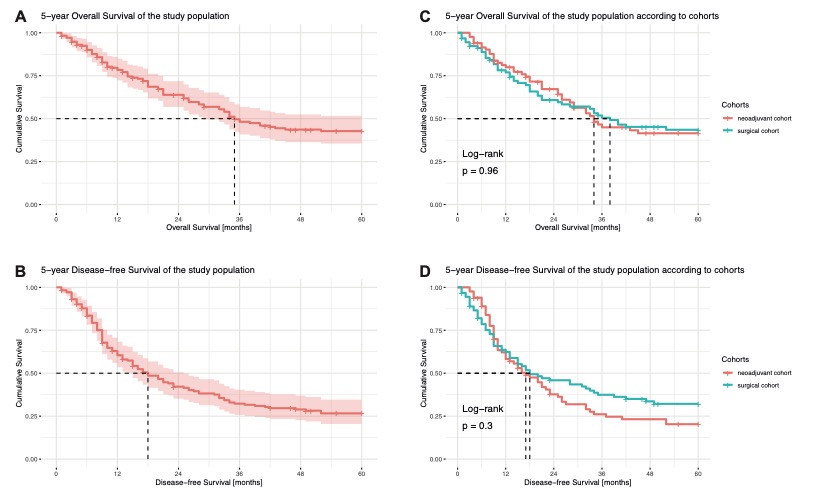


Figure S2: (A) Overall survival and (B) disease-free survival of the whole study population and (C, D) according to the corresponding sub cohort.

# Supplementary figure S3: Time-dependent change of marker expression

Figure S3: Distribution of (A) PD-L1 and (B) CD8 expression per year during the period of observation. Overall, there is no difference in distribution indicated by p > 0.05 using the Kruskal-Wallis test. The number of observations per year is indicated below each boxplot.

# Supplementary figure S4: Semi-automated lymphocyte detection


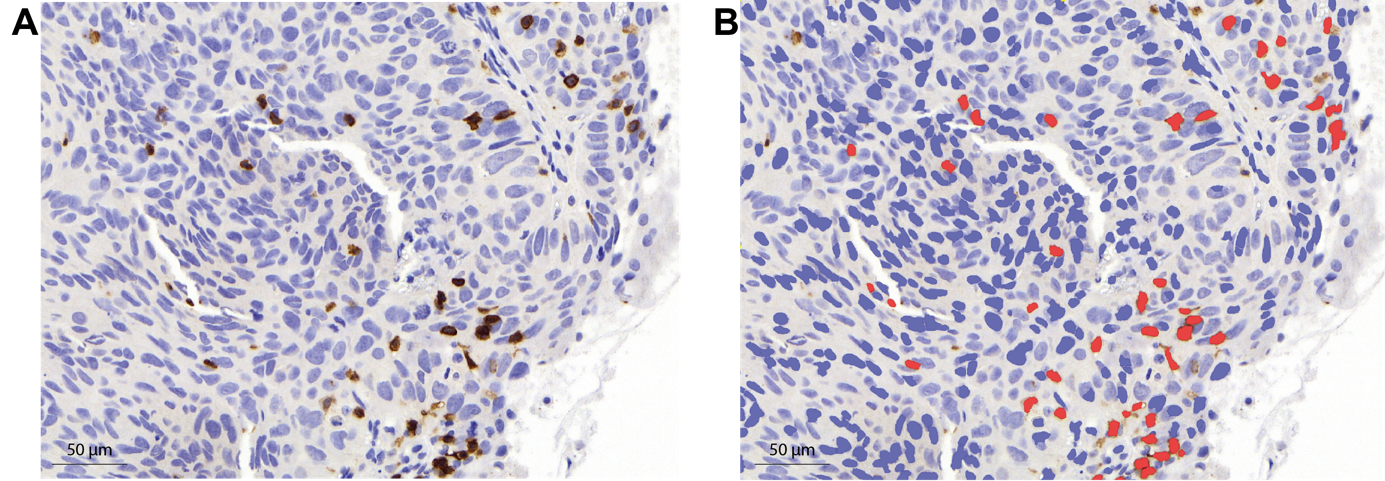


Figure S4: Lung tumor tissue sections immunohistochemically stained for CD8 were analyzed using QuPath. (A) The original image shows scattered lymphocytes stained in brown. (B) The same section with an overlay of the automatically detected and classified cells, CD8+ lymphocytes are marked in red.

# Supplementary figure S5: Association of smoking status with PD-L1 TPS


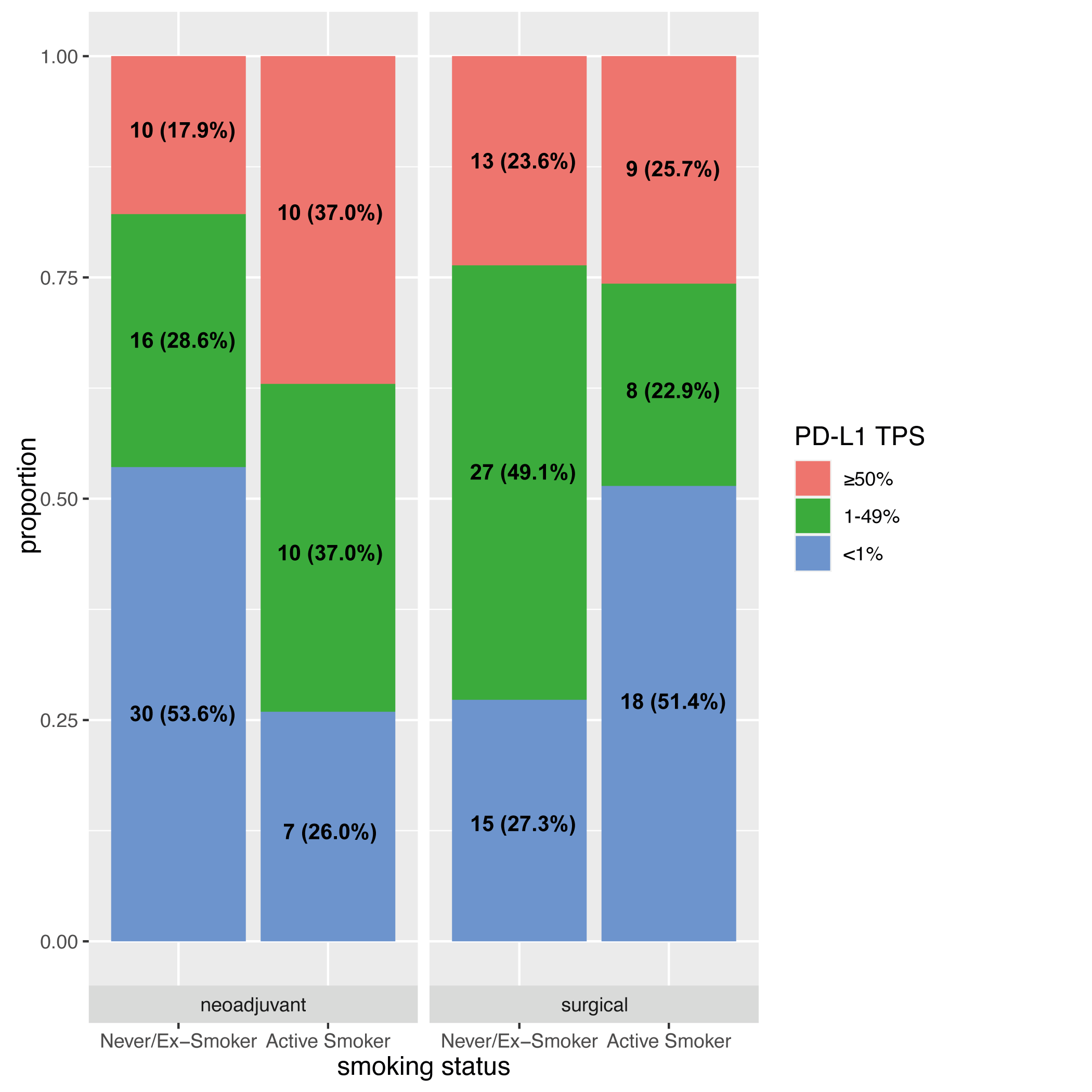


Figure S5: Association of smoking status with PD-L1 TPS according to the cohort. Using the original 3 tier classification, PD-L1 TPS was significantly associated with smoking status only in the neoadjuvant cohort. However, significantly more tumors were PD-L1 positive (≥1% PD-L1 TPS) in non-smokers in the surgical cohort.

# Supplementary figure S6: Change of raw PD-L1 TPS according to specimen


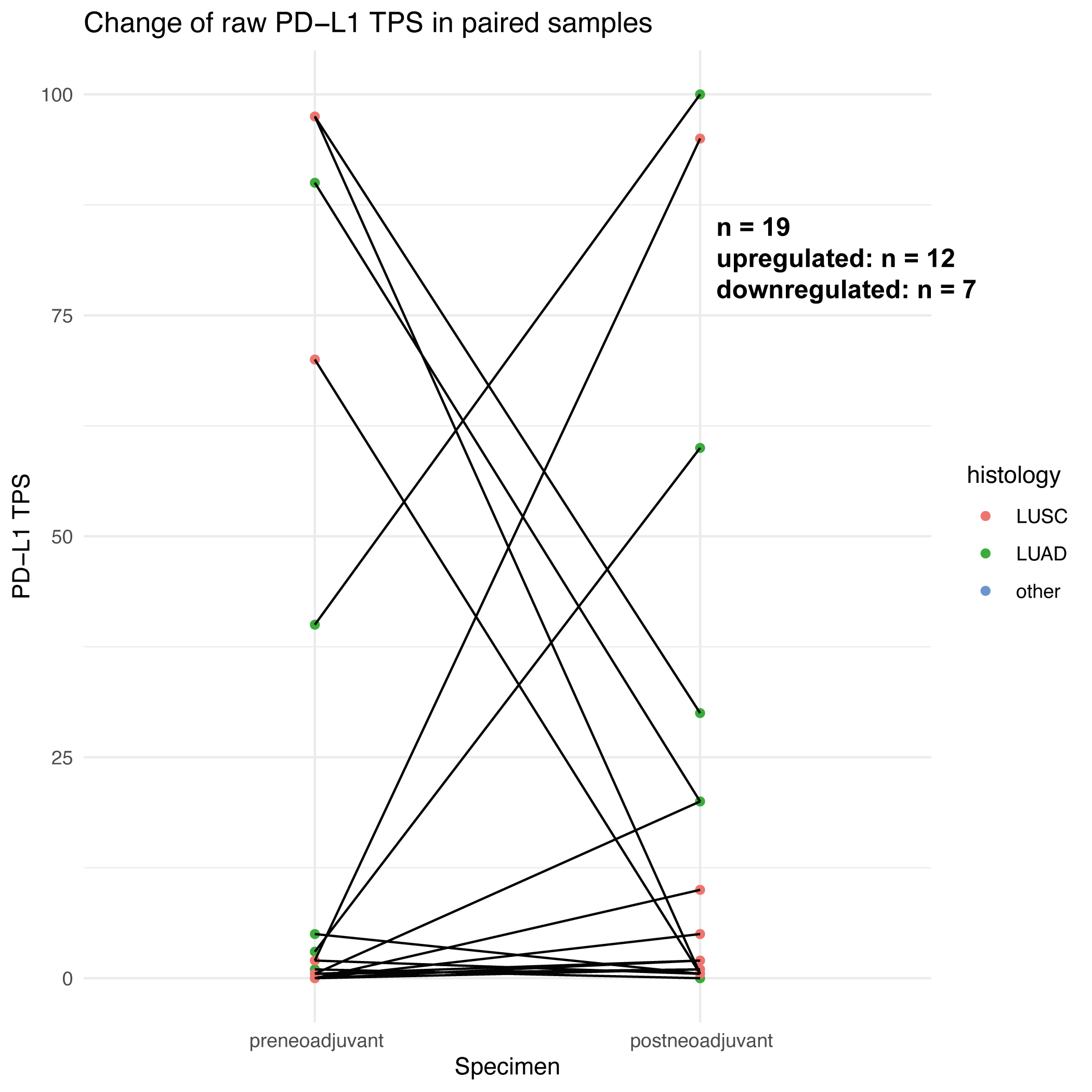


Figure S6: Change of raw (uncategorized) PD-L1 TPS in paired specimen. Only cases with changed PD-L1 TPS according to the clinical cut-offs (1%, 50%) were included for a clearer outline.

# Supplementary figure S7: CD8 TILs according to PD-L1 change (based on three tier classification of PD-L1)

*Figure S7: Change of CD8+ TILs density according to change of PD-L1 TPS in paired specimens (diagnostic biopsies versus resection specimens after neoadjuvant therapy). Significant decrease of CD8+ TILs (p = 0.012) was detected only in the group of unchanged PD-L1 expression, probably due to sufficient sample size in this group only.*

# Supplementary figure S8: Prognostic significance of PD-L1 in locally advanced resectable NSCLC

*Figure S8: PD-L1 has no prognostic significance in locally-advanced NSCLC (combination of neoadjuvant and surgical cohort). Kaplan Meier curves of (A, B, C) overall survival and (D, E, F) disease-free survival according to PD-L1 TPS (A, D) threefold, (B, E) 1% cut-off or (C, F) 50% cut-off.*

# Supplementary figure S9: CD8+ TILs are a prognostic factor in locally-advanced NSCLC

Figure S9: Patients with high CD8+ TILs densities have significantly better survival (combined neoadjuvant and surgical cohort). The density of CD8+ TILs is a prognostic factor for both (A) overall survival and (B) disease-free survival.

# Supplementary figure S10: Multivariable cox proportional hazard models for prognostic markers


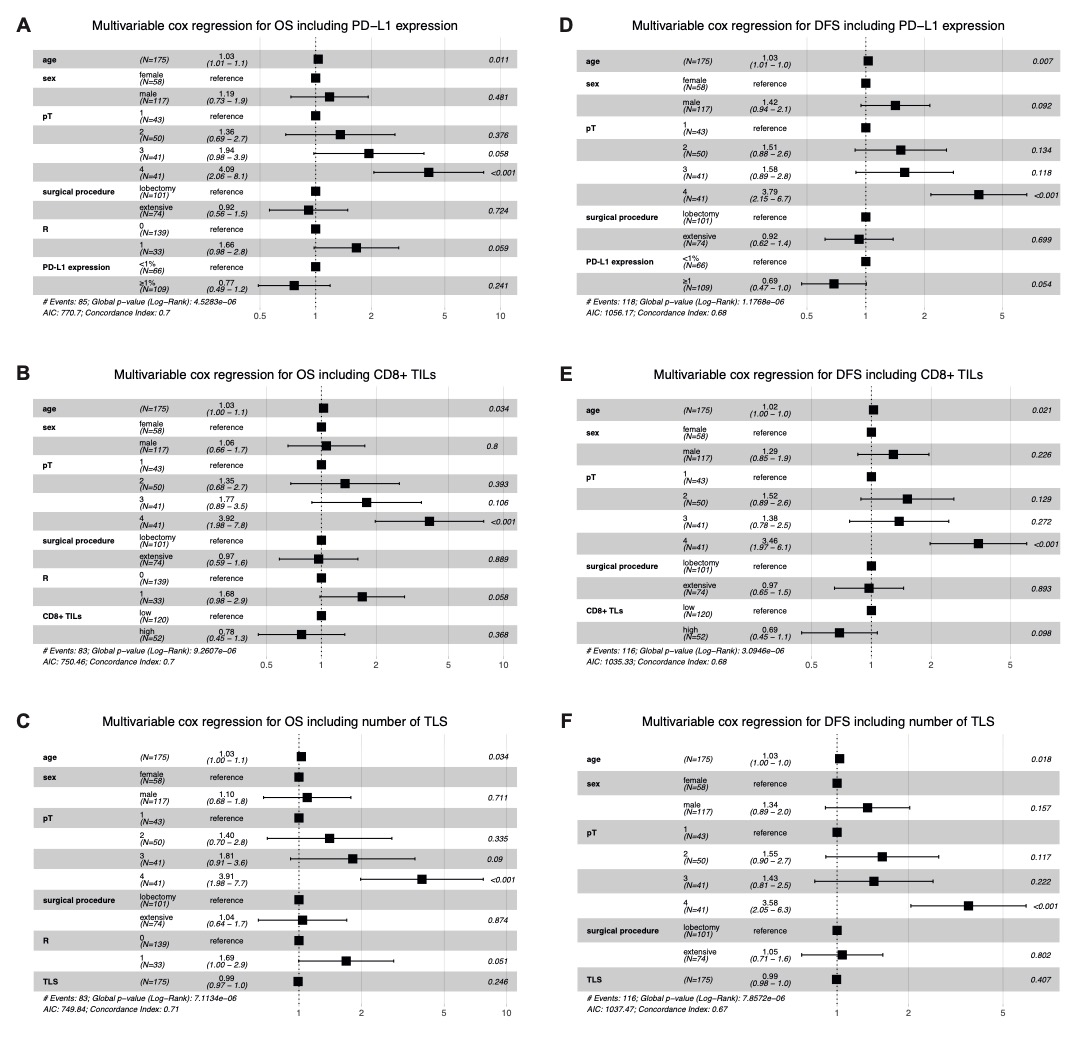


Figure S10: Forest plots integrating immune biomarkers in a multivariable cox proportional hazard model including all patients (combined neoadjuvant and surgical cohort) for (A-C) overall survival and (D-F) disease-free survival including (A, D) PD-L1 expression, (B, E) CD8+ TILs and (C, F) the number of tertiary lymphoid structures.

# Supplementary table S1: Comparison of manual and automatic TILs detection

Table 1: Comparison of manually and automatically counted infiltrating CD8+ lymphocytes.

# Supplementary table S2: Cases with changed PD-L1 expression after neoadjuvant therapy

Table S2: Patients with different PD-L1 expression (TPS) after neoadjuvant therapy. Cases with higher PD-L1 expression after neoadjuvant therapy are marked in green, whereas cases with a change to lower PD-L1 expression are marked in orange. Two cases (bold, larger font) showed a drastic decrease of PD-L1 expression from >95% (PID60) and 70% (PID109) to <1% respectively.

# Supplementary table S3: Association of change of PD-L1 expression with clinico-pathological parameters

Table S3: Assessment of potential associations of (A) sex, (B) histology, (C) presence or absence of major pathological response (MPR) and (D) change in CD8+ TILs density with change in PD-L1 TPS. PD-L1c: change of PD-L1 TPS (cut-offs 1% or 50%), LUAD: lung adenocarcinoma, CD8c: change of CD8+ TILs density. P-values are reported using Fisher’s exact test.
